# Malignant remodeling of the bone marrow microenvironment in acute myeloid leukemia: mechanisms and therapeutic implications

**DOI:** 10.3389/fonc.2026.1903019

**Published:** 2026-10-19

**Authors:** Wenhao Xu, Qing Han, Changrong Sun, Daqi Li

**Affiliations:** 1 Shandong Second Medical University, Weifang, China; 2 Jinan Central Hospital, Jinan, China

**Keywords:** acute myeloid leukemia, bone marrow microenvironment, immunotherapy, leukemic stem cells, metabolic reprogramming

## Abstract

Acute myeloid leukemia (AML) is an aggressive clonal myeloid malignancy characterized by the expansion of differentiation-arrested blasts. Treatment resistance, measurable residual disease, and relapse remain major causes of treatment failure. Experimental and clinical evidence indicates that the bone marrow microenvironment can protect heterogeneous AML-cell populations and can also sustain functionally defined or stem-enriched leukemic stem cell (LSC) populations through partly overlapping but non-identical mechanisms. Under physiological conditions, stromal, vascular, neural, and immune components coordinate hematopoietic stem-cell quiescence, retention, and regeneration. In AML, bulk leukemia cells remodel stromal, vascular, extracellular-matrix, metabolic, and immune compartments, whereas LSC-focused studies specifically implicate niche-dependent homing, quiescence, self-renewal, and persistence. This review therefore distinguishes evidence obtained in bulk AML cells from evidence obtained in functionally defined or stem-enriched LSC populations, compares conserved and disease-specific remodeling programs, and summarizes niche-directed therapeutic strategies.

## 1 Introduction

Acute myeloid leukemia (AML) is an aggressive hematologic malignancy characterized by the clonal expansion of differentiation-arrested myeloid cells (1, 2). Despite advances in risk-adapted therapy, allogeneic transplantation, and measurable residual disease-guided management, treatment resistance and relapse remain major clinical challenges (3, 4). The concept of an AML-propagating stem cell was first established by xenotransplantation studies showing that only a minor fraction of primary AML cells could initiate and serially propagate leukemia (5, 6), a paradigm subsequently elaborated in reviews of AML-microenvironment biology (7, 8). Accordingly, throughout this review, “AML cells” refers to bulk blasts or a heterogeneous leukemic population, whereas “LSCs” is used only when the cited study directly examined functional self-renewal or serial repopulation or a defined stem-enriched model; surface-marker expression alone is not treated as proof of LSC identity (7–9). Both intrinsic leukemic-cell programs and reciprocal interactions with the BMM contribute to disease persistence and therapeutic resistance (10–12).

The normal BMM is a multicellular ecosystem composed of mesenchymal stromal cells (MSCs), endothelial and osteolineage cells, immune cells, adipocytes, and neural elements (13–15). SCF- and CXCL12-producing stromal and vascular cells regulate HSC retention, quiescence, and activation (15–17). In AML, bulk leukemic cells can displace or reprogram normal niche components through neural, vascular, stromal, and adipocytic remodeling (12, 18–20). By contrast, LSC-focused studies demonstrate selective use of protected endosteal/perivascular regions and adhesion or chemokine pathways that support homing, quiescence, and self-renewal (6, 9, 20, 21).

Malignant BMM remodeling spans structural, stromal, metabolic, and immunological dimensions (10, 11, 22–24). Evidence obtained in bulk AML cells includes induction of stromal senescence, altered extracellular-matrix and vascular function, metabolic substrate use, and immune suppression (12, 18, 19, 23, 25–27). LSC-specific evidence is narrower and includes TET2-dependent homing/self-renewal, ALDH2-linked metabolic plasticity, and niche-protected quiescence (6, 9, 28, 29). This distinction is maintained throughout the manuscript rather than using “AML cells” and “LSCs” interchangeably.

This review first summarizes physiological BMM organization and then evaluates how bulk AML cells, LSC-enriched populations, MSCs, and immune cells participate in reciprocal niche remodeling (10–12, 22). Cross-disease comparisons are included only to identify potentially conserved niche programs and are explicitly labeled when the evidence derives from acute lymphoblastic leukemia (ALL), chronic myeloid leukemia (CML), or multiple myeloma rather than AML. Finally, we discuss how adhesion, metabolic support, stromal drug metabolism, immune suppression, and treatment-induced niche injury may be therapeutically targeted, while distinguishing preclinical proof-of-concept from AML-specific clinical evidence (12, 30–34).

## 2 Bone marrow microenvironment: from physiological homeostasis to leukemia-mediated malignant remodeling

### 2.1 Cellular composition of the normal bone marrow niche

The normal BMM comprises interconnected endosteal and perivascular niches rather than two fully isolated compartments (14, 17, 35–37). MSCs, endothelial cells, osteolineage cells, macrophages, adipocytes, and sympathetic nerves provide spatially and temporally regulated signals that control HSC quiescence, retention, mobilization, and lineage output (15, 35, 37–40). SCF and CXCL12 produced by stromal and endothelial populations are central to HSC maintenance, whereas vascular and neural signals contribute to HSC trafficking and regenerative activation (15–17, 41). These coordinated interactions are summarized in **Figure 1**.

**Figure 1:**
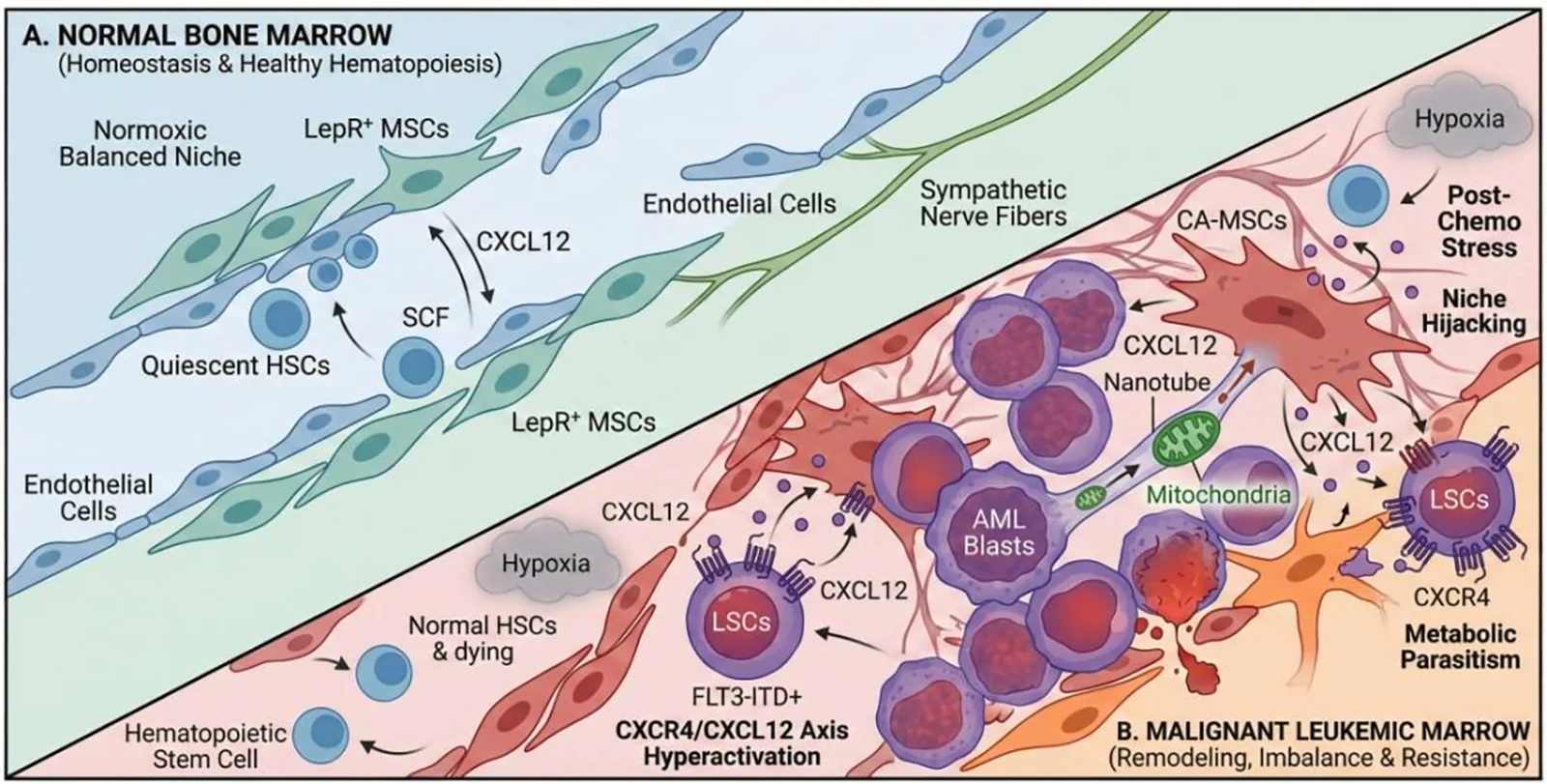
Comparison of the homeostatic and AML-remodeled bone marrow microenvironments. **(A)** Homeostatic bone marrow. HSCs are maintained by coordinated interactions with LepR+ MSCs, endothelial cells, sympathetic nerve fibers, SCF, and CXCL12. **(B)** Malignant leukemic marrow. AML blasts and LSC-enriched populations occupy and remodel the niche through CXCR4/CXCL12-dependent retention, stromal reprogramming, mitochondrial exchange, hypoxia-associated stress adaptation, and suppression of normal hematopoiesis. The image contrasts physiological hematopoietic homeostasis in panel **(A)** with malignant remodeling, imbalance, and treatment resistance in panel **(B)**; labels referring to LSCs denote only the stem-enriched mechanisms supported by LSC-focused studies.

### 2.2 Metabolic homeostasis and regulatory mechanisms of the normal niche

Low oxygen tension and restrained reactive oxygen species (ROS) production are characteristic of HSC-supportive marrow regions (42, 43). Quiescent HSCs rely predominantly on glycolytic metabolism, whereas activation and differentiation are accompanied by increased mitochondrial oxidative phosphorylation (OXPHOS) and ROS generation (43–45). Autophagy, mitophagy, amino-acid sensing, and proteostatic pathways help preserve HSC fitness during quiescence and stress (45, 46). Adipocytes and endothelial cells can additionally provide metabolic substrates and paracrine signals that influence hematopoietic-cell behavior (17, 38, 45).

Metabolic and epigenetic regulation are coupled within the normal niche because metabolites such as acetyl-CoA and amino-acid-derived intermediates influence chromatin-modifying reactions (43, 45). Sympathetic neural signaling contributes to circadian HSC mobilization through regulation of stromal CXCL12, and regulatory immune populations help limit inflammatory injury to the hematopoietic compartment (39–41, 47). During aging, injury, or regenerative stress, HSCs and niche cells undergo coordinated metabolic and transcriptional adaptation, illustrating the plasticity of the BMM (43, 45).

AML disrupts this homeostatic organization through several experimentally distinct processes. Bulk AML cells can induce sympathetic neuropathy, vascular/endosteal remodeling, stromal senescence, and loss of supportive adipocytic or hematopoietic niches (12, 18, 19, 23). LSC-focused studies, in contrast, show that stem-enriched AML populations can preferentially home to protected marrow regions and use niche signals to maintain quiescence, self-renewal, and treatment persistence (6, 9, 20, 21, 29). These complementary processes provide a mechanistic basis for impaired normal hematopoiesis and for microenvironment-mediated resistance.

## 3 Malignant remodeling of the BMM by leukemia cells and leukemic stem cells

The interaction between AML and the BMM is bidirectional: leukemic populations alter stromal, vascular, neural, and immune compartments, and the remodeled niche feeds back through adhesion, soluble factors, metabolic support, and drug protection (10–12, 22). **Figure 2** summarizes mechanisms supported in bulk AML cells together with mechanisms demonstrated in LSC-enriched or functionally defined populations; the two evidence categories are labeled separately.

**Figure 2:**
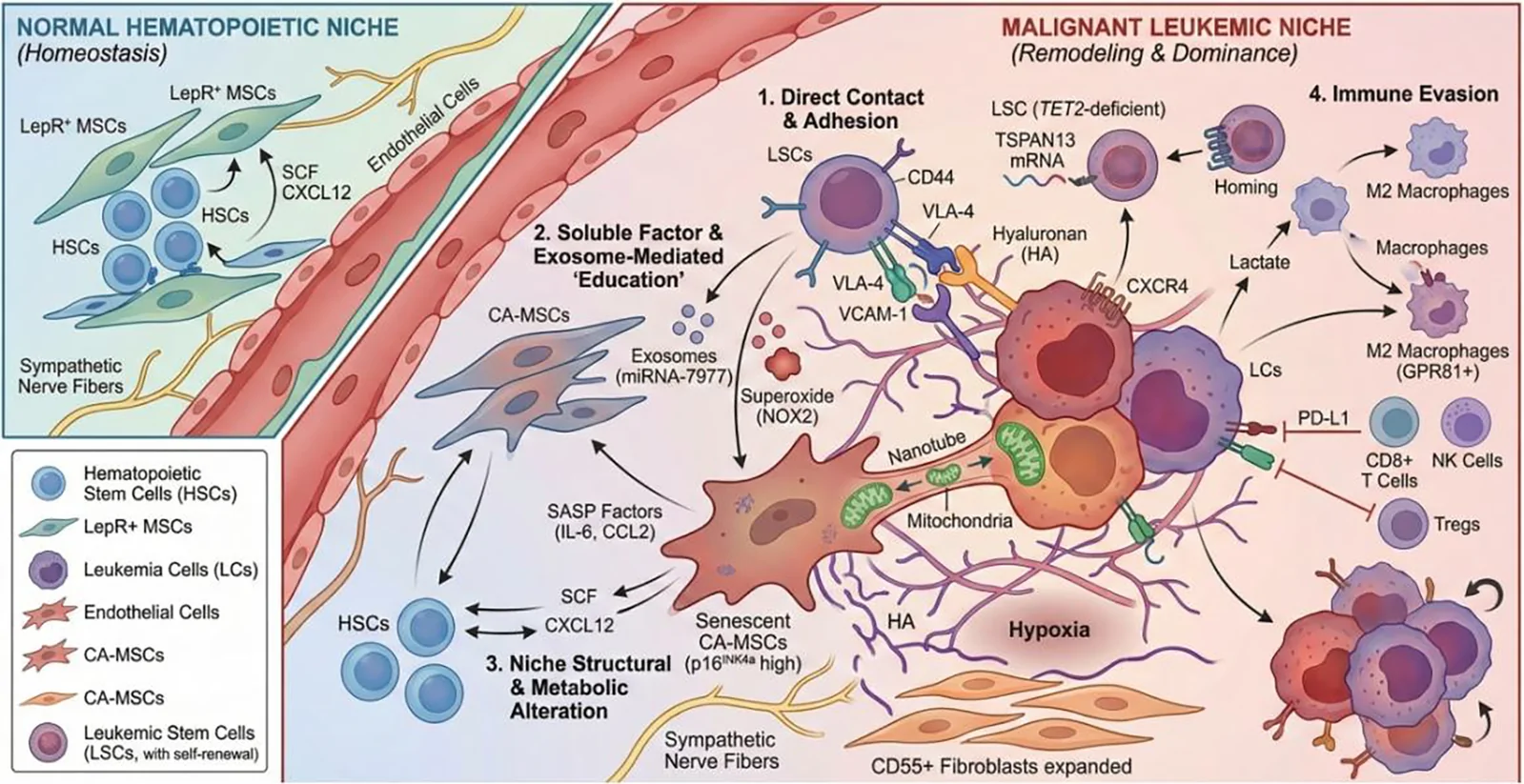
Mechanisms of bone marrow microenvironment remodeling by bulk AML cells and LSC-enriched populations. The left inset depicts a homeostatic HSC niche supported by LepR+ MSCs, endothelial cells, sympathetic nerves, SCF, and CXCL12. The malignant niche summarizes four interacting programs: (1) direct adhesion and retention through CD44, VLA-4/VCAM-1, and TET2-TSPAN13-CXCR4 signaling; (2) soluble-factor and extracellular-vesicle-mediated stromal education; (3) structural and metabolic remodeling, including p16INK4a-associated stromal senescence, CD55+ fibroblast expansion, hypoxia, and mitochondrial transfer; and (4) immune evasion through checkpoint and lactate-associated pathways. Mechanisms demonstrated in bulk AML cells are distinguished in the text from mechanisms established in LSC-focused models.

### 3.1 Leukemia-cell- and LSC-mediated structural and functional remodeling of the bone marrow microenvironment

Bulk AML cells and stem-enriched/preleukemic populations remodel the BMM through related but experimentally distinguishable mechanisms (**Figure 2**). AML cells can reprogram MSC bioelectric signaling through CaV1.2-dependent mechanisms and can induce p16INK4a-driven stromal senescence with a pro-leukemic secretory phenotype (23, 48). Preleukemic stem/progenitor populations have been associated with expansion of CD55+ fibroblasts and reduced collagen expression; this finding should not be conflated with evidence obtained in established LSCs (49). Niche TWIST1 supports normal hematopoiesis, and its loss in the stromal compartment impairs HSC-supportive signals; the cited study does not establish leukemia-cell TWIST1 as the causal niche factor (50). In a distinct LSC-focused study, TET2-mediated mRNA demethylation regulated TSPAN13 expression and the CXCR4/CXCL12 axis, thereby controlling LSC homing and self-renewal (29). Classical AML models further demonstrate leukemia-induced sympathetic neuropathy, endosteal vascular remodeling, and adipocytic-niche disruption (12, 18, 19).

### 3.2 Microenvironmental support of AML-cell and LSC survival, state transitions, and drug resistance

The remodeled BMM supports both bulk AML cells and LSC-enriched populations, but the relevant mechanisms depend on the experimental system. In bulk AML cells, CD44 engagement increases VLA-4 avidity and strengthens VCAM-1-dependent adhesion and prosurvival signaling (51). In LSC-focused models, TET2-dependent TSPAN13 expression enhances CXCR4-mediated homing and self-renewal, and the ALDH2-PKCδ-SHMT2 axis supports metabolic plasticity and chemotherapy evasion (28, 29). ALDH3A2 protects AML cells from oxidative death and ferroptosis-inducing stress, but that study did not define the protected population as LSCs (52). CX43-mediated mitochondrial transfer preserved stem-like properties in the KG-1a model, whereas primary AML studies have demonstrated chemotherapy-associated mitochondrial transfer to bulk leukemic cells (26, 53). Collectively, these data support context-dependent maintenance of quiescence, survival, or proliferation rather than a single uniform “LSC-supportive” niche program (6, 9, 29).

Microenvironment-mediated resistance has also been demonstrated in molecularly defined AML-cell populations. In FLT3-mutant AML, BTK-dependent autophagy contributes to FLT3-inhibitor resistance, and longitudinal profiling has shown stepwise niche-associated evolution to gilteritinib resistance, including early dependence on AURKB (54, 55). TGF-βR-SMAD3 signaling can increase DNA double-strand-break repair and PARP-inhibitor resistance in marrow-based leukemia models; because that evidence is not LSC-specific, it is described here as a leukemia-cell resistance mechanism rather than an LSC mechanism (56).

### 3.3 Cross-disease comparison: conserved and disease-specific programs of bone marrow microenvironment remodeling

A cross-disease comparison is useful only when the source malignancy is stated explicitly. Across marrow-based hematologic cancers, recurrent themes include chemokine- or integrin-mediated retention, stromal-cell reprogramming, treatment-induced adipogenic changes, and metabolic exchange (51, 57–61). However, the participating cell populations, dominant cytokines, oncogenic context, and therapeutic consequences differ substantially; therefore, observations from ALL, CML, or multiple myeloma should not be presented as direct evidence for AML.

In B-ALL, MSC conversion toward cancer-associated fibroblast-like states and therapy-enriched adipogenic progenitors support migration and drug resistance through SDF-1/CXCR4 and IL-7/VCAM1 pathways (57, 58). In T-ALL, dexamethasone-induced adipocytes protect residual cells through a CXCL13-associated program (62). These lymphoid-leukemia findings emphasize treatment-dependent stromal plasticity but involve lineage-specific cytokine programs that have not been established as dominant mechanisms in AML.

In CML, TNF-α expands 6C3+ mesenchymal precursors that promote CML LSC growth through CXCL1/CXCR2, while direct stromal contact transfers metabolic proteins and contributes to imatinib resistance in CML cells (59, 60). These mechanisms arise in a chronic BCR::ABL1-driven disease context and therefore differ from the acute clonal and treatment dynamics of AML.

Multiple myeloma is included only as a marrow-resident plasma-cell malignancy comparator, not as a leukemia subtype. Its reciprocal interaction with marrow adipocytes produces adipocyte senescence and metabolic dysfunction that supports dexamethasone resistance (61). This comparison illustrates a conserved principle—bidirectional metabolic remodeling—while also highlighting myeloma-specific dependence on the bone and adipocyte niche.

For AML, the most directly supported nodes include CaV1.2-dependent stromal reprogramming, p16INK4a-driven senescence, CD44/VLA-4-mediated adhesion, TET2-TSPAN13-CXCR4-dependent LSC homing, ALDH-mediated redox control, and stage-specific gilteritinib resistance (23, 28, 29, 48, 51, 52, 54, 55). Accordingly, AML-directed translation should prioritize validation of these AML-derived mechanisms, whereas CAF, IL-7/VCAM1, CXCL13/Notch1, CXCL1/CXCR2, and adipocyte-senescence pathways from other malignancies should be treated as comparative hypotheses rather than established AML targets (57–59, 61, 62). This disease-specific framing clarifies that the final therapeutic discussion in this section refers to AML unless another malignancy is explicitly named.

## 4 Functional remodeling and bidirectional regulation of bone marrow mesenchymal stem cells in the AML microenvironment

### 4.1 Homeostatic versus AML-associated MSCs: phenotypic and functional reprogramming

Homeostatic MSC populations support HSC retention and lineage output through SCF, CXCL12, extracellular-matrix organization, and balanced osteogenic/adipogenic differentiation (14–17, 37). AML-associated MSCs are not a single uniform cell state; patient- and model-dependent studies report senescence, altered cytokine secretion, ID1- or TWIST1-associated transcriptional changes, impaired osteogenesis, enhanced adipogenesis, and reduced support of normal hematopoiesis (23, 63–66). Functionally, these remodeled stromal populations can promote AML-cell survival, retention, metabolic adaptation, and drug resistance through direct contact, soluble mediators, mitochondrial transfer, and local drug metabolism (51, 53, 67, 68). **Figure 3** contrasts homeostatic support functions with the disease-associated reprogramming and feedback mechanisms documented in AML models.

**Figure 3:**
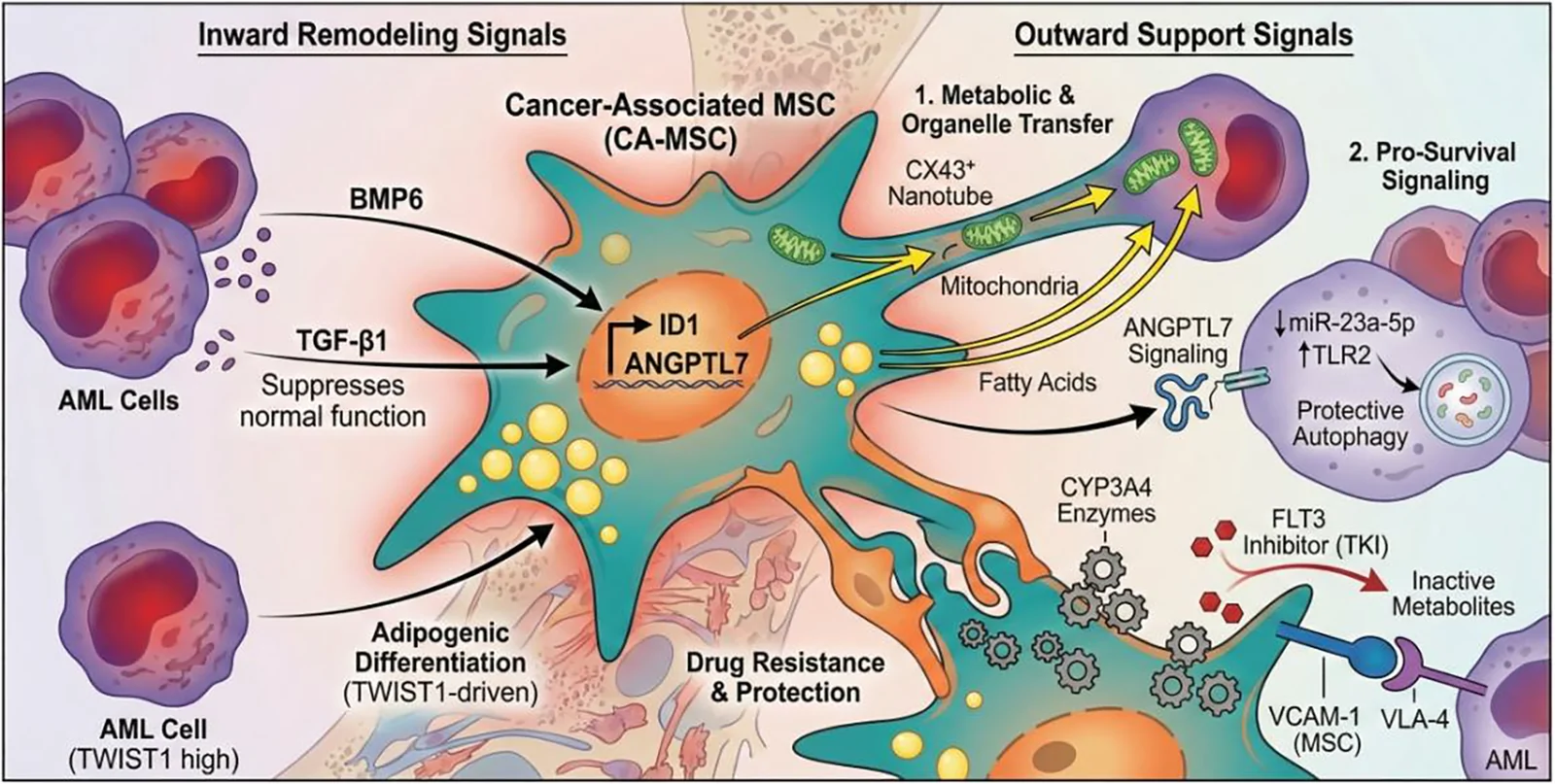
Bidirectional reprogramming between AML cells and bone marrow mesenchymal stromal cells. AML-to-MSC signals include BMP6-ID1-ANGPTL7 signaling, TGF-β1-mediated loss of hematopoietic support, and TWIST1-associated adipogenic reprogramming. MSC-to-AML feedback includes CX43-mediated mitochondrial transfer in a KG-1a stem-like model, fatty-acid support, CYP3A4-mediated inactivation of FLT3 inhibitors, miR-23a-5p/TLR2-dependent protective autophagy, and VCAM-1/VLA-4 adhesion. The image depicts model-specific mechanisms and does not imply that every pathway is present in all primary AML samples or all LSCs.

### 4.2 Bidirectional regulatory mechanisms between AML cells and remodeled MSCs

AML cells can reprogram MSCs through multiple non-cell-autonomous pathways. AML-derived BMP6 induces ID1 in MSCs; ID1 interacts with RNF4, stabilizes SP1, and increases ANGPTL7 production, thereby supporting AML progression (64). TWIST1-high MDS/AML cells can redirect MSC differentiation toward an adipogenic, leukemia-supportive state, while TGF-β1 contributes to functional inhibition of hematopoietic support by MSCs (65, 66). Conversely, remodeled MSCs downregulate miR-23a-5p in AML cells to activate TLR2-dependent protective autophagy, and adipogenic reprogramming is associated with enhanced chemoresistance (63, 68). MSC-expressed CYP3A4 can metabolically inactivate FLT3 inhibitors, whereas VCAM-1/VLA-4 adhesion activates prosurvival signaling and increases AML-cell retention (51, 67). CX43-mediated mitochondrial transfer maintained stem-like properties in KG-1a cells, and TGF-βR-SMAD3 signaling enhanced PARP-inhibitor resistance in marrow leukemia models; these findings are therefore identified as model-specific rather than generalized to all primary LSCs (53, 56).

### 4.3 Comparative MSC programs in ALL and CML

In B-ALL, leukemia-induced conversion of MSCs toward cancer-associated fibroblast-like states can enhance migration and drug resistance through SDF-1/CXCR4 signaling (57). Distinct adipogenic progenitor populations support B-ALL through IL-7 and VCAM1, and GREM1-deficient MSCs promote an adipose niche through BMP/SMAD signaling (58, 69). In T-ALL, dexamethasone-induced adipocytes can attract and protect residual cells through a CXCL13-associated program (62). In CML, TNF-α-expanded mesenchymal precursors support CML LSC growth through CXCL1/CXCR2, and stromal transfer of metabolic proteins contributes to imatinib resistance in CML cells (59, 60). These examples identify disease-specific MSC programs and should not be interpreted as direct AML evidence.

## 5 Immune remodeling and metabolic competition jointly drive immunosuppression in the AML bone marrow microenvironment

In the AML BMM, immune dysfunction involves multiple cell types rather than a single LSC-specific mechanism (**Figure 4**). Bone marrow T cells from patients with AML can exhibit reversible functional suppression, increased checkpoint expression, and defective immune-synapse formation (70). AML-cell IDO1 expression is associated with altered B-cell subset composition, including fewer naïve B cells and more regulatory B cells (71). CML single-cell studies have identified expansion and clonal diversity of terminal effector T-cell subsets; this is presented as CML evidence and not as proof of a universal AML program (72). Single-cell analysis after donor lymphocyte infusion has associated specific CD8+ ZNF683+ populations with therapeutic response, but the data support an association rather than the claim that one subset alone determines outcome (73).

**Figure 4:**
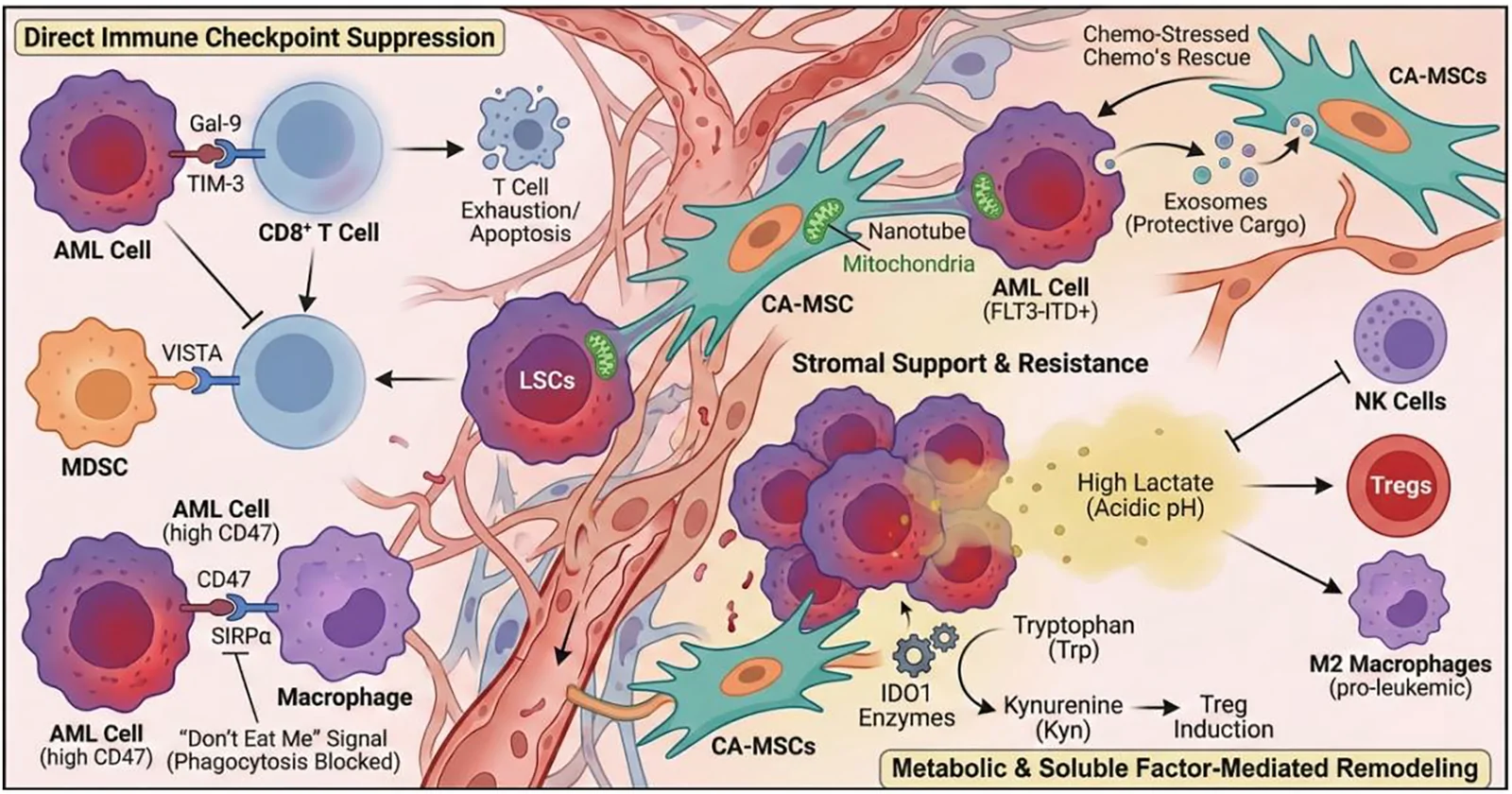
Immune and metabolic remodeling of the AML bone marrow microenvironment. Checkpoint-mediated suppression includes CD47-SIRPα inhibition of macrophage phagocytosis, TIM-3-associated LSC immune escape, and PD-1-associated T-cell dysfunction. Metabolic and soluble-factor mechanisms include IDO1-associated immune dysregulation and lactate-GPR81-dependent polarization of macrophages toward an immunosuppressive phenotype. Inflammatory remodeling includes ANXA5-loss-associated activation of the COX2/PGE2 axis. The schematic integrates AML-cell, LSC-specific, stromal, and immune-cell evidence; the corresponding population is specified in the main text.

Immune suppression in AML is also linked to metabolic competition and inflammatory remodeling (23, 74). High glycolytic activity and oxygen consumption by leukemic cells can restrict substrates available to effector immune cells, while lactate accumulation promotes GPR81-dependent polarization of macrophages toward an immunosuppressive phenotype (27, 75). Loss of ANXA5 increases inflammatory conditioning and activates the COX2/PGE2 axis, which supports AML progression and can alter the activity of surrounding immune cells (25). These findings concern bulk AML-cell and microenvironmental interactions unless an LSC-specific population is explicitly identified.

Disrupting stromal protection may improve immune-mediated cytotoxicity, but the disease model must be specified. VLA-4 blockade sensitized leukemic and myeloma cells to CD3-redirection therapy in preclinical marrow models; this provides a cross-disease proof-of-concept rather than AML-specific clinical evidence (30). In an AML model, activation of the NLRP3 inflammasome in bone marrow dendritic cells increased IL-1β, promoted Th1/IFN-γ responses, and exerted antileukemic activity (76). Together, these studies support further AML-specific evaluation of adhesion blockade, metabolic intervention, and immune reactivation.

## 6 Treatment strategies

Because the BMM contributes to AML-cell survival, LSC persistence, and treatment resistance, therapeutic development increasingly combines leukemia-directed agents with strategies that disrupt protective niche interactions (10–12). Current approaches include blockade of adhesion or homing, extracellular-matrix disruption, marrow-targeted nanodelivery, induction of alternative cell-death programs, immune reactivation, and restoration of normal hematopoietic support (30–34, 77). The evidence base ranges from *in vitro* and animal studies to selected clinical studies, as summarized in **Table 1**.

**Table 1:** Summary of therapeutic strategies targeting the AML bone marrow microenvironment.

Therapeutic strategy	Targeted pathway/cell population	Mechanism of action	Development stage
LOXL2 inhibitor-loaded yolk-shell nanoparticles (31)	Extracellular matrix (collagen scaffold)	Disrupts collagen architecture, detaches AML cells from matrix protection, and increases chemotherapy sensitivity	Preclinical (*in vitro* and *in vivo*)
CXCR4 antagonist plus chemotherapeutic/siRNA co-delivery system (32)	CXCR4/CXCL12 axis; LSC-enriched AML populations	Reduces marrow homing/retention, inhibits mitophagy, and decreases stemness-related and relapse-associated phenotypes	Preclinical
Bisphosphonate-modified C-Myb/Survivin nanocomposite (78)	MLL-AF9-rearranged AML cells	Provides marrow-directed delivery and suppresses C-Myb/Survivin signaling; selective activity against functionally defined LSCs was not established	Preclinical (including PDX models)
CD123-targeted EZH2-siRNA aptamer nanoparticles (79)	CD123+ MLL-AF9-rearranged AML cells	Delivers EZH2 siRNA and inhibits leukemic-cell proliferation through epigenetic targeting	Preclinical
SMAC mimetics plus HDAC inhibitors (80)	RIPK1-dependent necroptosis in ALL	Bypasses apoptotic defects and induces necroptosis; included as a cross-disease proof-of-concept, not AML evidence	Preclinical (ALL models)
BET protein degrader ARV-825 (33)	AML cells, stem/progenitor populations, and stromal protection	Degrades BET proteins, suppresses MYC-associated transcription, and reduces microenvironment-mediated protection	Preclinical
Arsenic trioxide (81)	AML blasts and stromal cytokine programs	Directly reduces AML blasts and reprograms leukemia-supportive stromal signaling	Preclinical
N-acetyl-L-cysteine (34)	Bone marrow endothelial cells and oxidative stress after haploidentical HSCT	Protects endothelial cells and promotes hematopoietic reconstitution; not an AML-specific niche-targeting trial	Phase 3 transplantation trial
VLA-4 integrin blockade (30)	VLA-4/VCAM-1 adhesion in leukemic and myeloma models	Disrupts stromal protection and enhances CD3-redirection-mediated cytotoxicity	Preclinical; cross-disease models
NLRP3 inflammasome activation in bone marrow dendritic cells (76)	IL-1β/Th1/IFN-γ immune axis in AML	Promotes Th1 differentiation and antileukemic immune activity	Preclinical AML model

### 6.1 Blocking adhesion/retention and disrupting the extracellular matrix barrier

AML cells can be retained in protective marrow regions through chemokine receptors, integrins, and extracellular-matrix interactions (12, 51). LOXL2-inhibitor-loaded yolk-shell nanoparticles disrupted the collagen scaffold and increased chemotherapy sensitivity in preclinical AML models (31). A marrow-responsive polymeric drug/siRNA system that co-targeted CXCR4 and mitophagy reduced LSC homing, stemness, and relapse-related phenotypes in preclinical AML models (32). These studies support combined physical mobilization and leukemia-cell killing, but clinical efficacy remains to be established.

### 6.2 Nanomedicine-based precise bone marrow targeting and epigenetic regulation

Nanodelivery systems can increase marrow exposure and reduce off-target distribution, although most AML applications remain preclinical (77). A bisphosphonate-modified nanocomposite targeting the C-Myb/Survivin axis suppressed MLL-AF9-rearranged AML in cell, animal, and patient-derived xenograft models; the cited study does not establish selective activity against functionally defined LSCs (78). CD123-targeted aptamer nanoparticles delivering EZH2 siRNA inhibited MLL-AF9-rearranged AML cells through epigenetic targeting, again without requiring an LSC-specific interpretation (79). These examples illustrate precise marrow or leukemia-cell targeting rather than proof of universal LSC eradication.

### 6.3 Inducing novel cell death modalities and microenvironment reprogramming

Alternative cell-death programs may help bypass apoptosis resistance, but evidence from other diseases must be labeled. The combination of SMAC mimetics and HDAC inhibitors induced RIPK1-dependent necroptosis in ALL models and should be considered a cross-disease proof-of-concept rather than AML evidence (80). In AML, the BET degrader ARV-825 targeted leukemic stem/progenitor cells and reduced stromal protection in preclinical systems (33). Arsenic trioxide has also been reported to reprogram the AML BMM while directly reducing AML blasts (81). These approaches remain investigational and require evaluation of toxicity to normal hematopoietic and stromal compartments.

### 6.4 Promoting microenvironment restoration and hematopoietic reconstitution

Restoration of normal hematopoiesis is an important complement to leukemia eradication (82, 83). In a phase 3 trial after haploidentical hematopoietic stem cell transplantation, prophylactic N-acetyl-L-cysteine improved endothelial-cell function and hematopoietic reconstitution; this was a transplantation study and not an AML-specific niche-targeting trial (34). Co-culture systems combining leukemia cells with stromal cells or adipocytes provide preclinical platforms for identifying interventions that preserve normal niche function while disrupting leukemia-supportive interactions (84).

### 6.5 Impact of conventional AML therapies on the bone marrow microenvironment

Conventional AML therapy affects both leukemic cells and nonmalignant niche components (12, 23). AML itself and cytotoxic stress can promote stromal senescence and a senescence-associated secretory phenotype containing factors such as IL-6 and CCL2 (12, 23). During complete remission, remodeling and repair of bone marrow endothelial progenitor cells are associated with recovery of normal hematopoiesis, highlighting the importance of vascular restoration after treatment (82, 83). Chemotherapy-associated adipogenic differentiation of MSCs can provide metabolic support and increase resistance of residual AML cells (63). In FLT3-mutant AML-cell populations, hypoxia/autophagy-associated signaling and stepwise microenvironmental adaptation contribute to FLT3-inhibitor resistance (54, 55).

Venetoclax-based therapy has both leukemia-directed and microenvironmental effects. Preclinical and clinical studies established that BCL-2 inhibition can preferentially disrupt OXPHOS in quiescent LSCs and that venetoclax plus azacitidine can target LSC energy metabolism in AML patients (85, 86). More recent work indicates that venetoclax/azacitidine may also alter sinusoidal endothelial integrity and the composition or activation state of immune-cell subsets (87, 88). Macrophage-phagocytosis enhancement has been proposed for the combination regimen, but the available report is preliminary and should be interpreted cautiously (89). Conversely, stromal signaling can increase alternative anti-apoptotic proteins and metabolic flexibility in AML cells, contributing to venetoclax resistance (26, 90, 91).

## 7 Conclusion and perspectives

AML cells and the BMM undergo dynamic, bidirectional co-evolution, but bulk AML cells and LSCs should not be treated as interchangeable experimental entities (9–12). Bulk leukemic populations can remodel vascular, neural, stromal, metabolic, and immune compartments, whereas LSC-focused studies more specifically support niche-dependent homing, quiescence, self-renewal, and persistence (6, 9, 20, 21, 28, 29). The resulting niche can protect residual disease through adhesion, metabolic exchange, stromal drug metabolism, senescence-associated signaling, and immune suppression (22, 23, 27, 51, 67).

Niche-directed approaches, including adhesion blockade, marrow-targeted nanomedicine, metabolic intervention, and immune reactivation, have produced encouraging preclinical results, but translation is limited by model dependence and the cellular and spatial heterogeneity of the human BMM (11, 12, 77, 92). We therefore propose five priorities: spatially resolved mapping of AML-cell and LSC states across marrow subniches; functional validation of putative LSC-specific mechanisms; humanized organoid or organ-on-chip models; rational scheduling of niche-directed agents with chemotherapy or immunotherapy; and strategies that restore normal vascular and stromal function after remission. These priorities are intended to prevent overgeneralization across diseases and experimental populations while accelerating clinically testable AML-specific interventions.
